# Development of a risk prediction model for the first occurrence of thrombosis in patients with OAPS

**DOI:** 10.3389/fimmu.2024.1459548

**Published:** 2024-10-04

**Authors:** Jie Gao, Yan Zheng, Zhuo Wang, Junfeng Jia, Jian Wan, Qing Han, Xi Zheng, Renli Liu, Zhaohui Zheng, Kaichun Wu, Ping Zhu

**Affiliations:** ^1^ Department of Clinical Immunology, Xijing Hospital, The Fourth Military Medical University, Xi’an, China; ^2^ State Key Laboratory of Holistic Integrative Management of Gastrointestinal Cancers and National Clinical Research Center for Digestive Diseases, Xijing Hospital of Digestive Diseases, The Fourth Military Medical University, Xi’an, China

**Keywords:** antiphospholipid syndrome, thrombosis, obstetric antiphospholipid syndrome, antiphospholipid antibodies, nomogram

## Abstract

**Objectives:**

The aim of this study is to assess the risk factors associated with thrombotic events in obstetric antiphospholipid syndrome (OAPS) patients and to develop a predictive model specifically tailored to predict the risk of postpartum thrombosis in OAPS patients without prior thrombotic events. This research seeks to enhance clinician’s awareness regarding the postpartum care and monitoring of OAPS patients.

**Methods:**

A retrospective study was conducted at the First Affiliated Hospital of the Fourth Military Medical University including 269 consecutive inpatients diagnosed with antiphospholipid syndrome (APS) from July 1, 2008 to July 31, 2022. All participants met the 2006 Sydney APS classification criteria or the “non-criteria OAPS classification”. Out of 98 candidate clinical and laboratory parameters considered, 40 potential variables were selected for analysis based on expert opinion. The logistic regression mode with the Least Absolute Shrinkage and Selection Operator (LASSO) were used to identify optimal predictive characteristics. All samples were included in the model building and a nomogram was generated based on these characteristics. The differentiation, calibration, and clinical utility of the predictive model were evaluated using the area under the curve (AUC), calibration curve, and decision curve analysis. The model was also validated by a 1000 bootstrap tests.

**Results:**

126 patients with OAPS were enrolled, and a total of 89 OAPS patients who had never experienced thrombosis were retrospectively analyzed. After 3 years follow-up, 32.58% of the patients (29/89) developed thrombosis. In order to create, LASSO logistic regression identified three optimal variables: the platelet count less than 125×109/L, more than one positive aPLs (antiphospholipid antibody), and the use of low molecular weight heparin (LMWH) or low dose aspirin (LDA) after delivery. A predictive model was conducted using these three predictive indicators for patients with OAPS who experience thrombosis for the first-time. This prediction model has good distinction, good calibration, and fair clinical practicality.

**Conclusion:**

Our model has good predictive ability in assessing the risk of thrombosis in patients with OAPS without prior thrombotic events. This model is easy to predict, has good discriminability and calibration, and can be utilized as a routine tool for thrombus screening in OAPS patients.

## 1 Introduction

Antiphospholipid syndrome (APS), including thrombotic antiphospholipid syndrome (TAPS) and obstetric antiphospholipid syndrome (OAPS), is an autoimmune disease associated with recurrent thrombosis and morbid pregnancy. It is characterized by concomitant persistence of antiphospholipid antibodies at serum levels of moderate to high titer (1). In severe circumstances, thrombosis can lead to disability or even death for a patient, severely impairing their quality of life. Some patients with obstetric antiphospholipid syndrome may develop thrombosis sometime after delivery. However, there is currently no reliable method to identify these high-risk populations.

Currently, the GAPSS (Global Antiphospholipid Syndrome Score) and aGAPSS (adjusted Global Antiphospholipid Syndrome Score) are mainly used for prediction of thrombosis risk in patients with antiphospholipid syndrome (2). Because the GAPSS score includes an antibody to PS/PT (phosphatidylserine/prothrombin), which is infrequently used in laboratory testing, the aGAPSS score is more commonly used clinically. On the other hand, little is known about how well aGAPSS predicts thrombosis in patients with OAPS (no history of thrombotic events). Several studies have investigated predictors of APS prognosis, but risk factors vary greatly in study designs, patient selection criteria and aPLs profiles (3). Platelets play an important role in the pathogenesis of antiphospholipid syndrome, interacting with the components of the coagulation system and the immune system, such as neutrophils and lymphocytes. The 2023 ACR/EULAR Antiphospholipid Syndrome Guideline has already included clinical manifestations such as thrombocytopenia as one of the inclusion criteria in the APS classification (4).

Our study evaluated the risk factors for thrombosis in OAPS patients with a history of obstetric events only (no previous thrombotic events). We aimed to develop a prediction model to predict the risk of thrombosis in these patients. The goal is to emphasize the importance of postpartum follow-up in OAPS patients for clinicians. At the same time, this model was compared with the aGAPSS score, and its predictive value for thrombosis was assessed.

## 2 Method

### 2.1 Patient assessment and data collection

**Figure 1:**
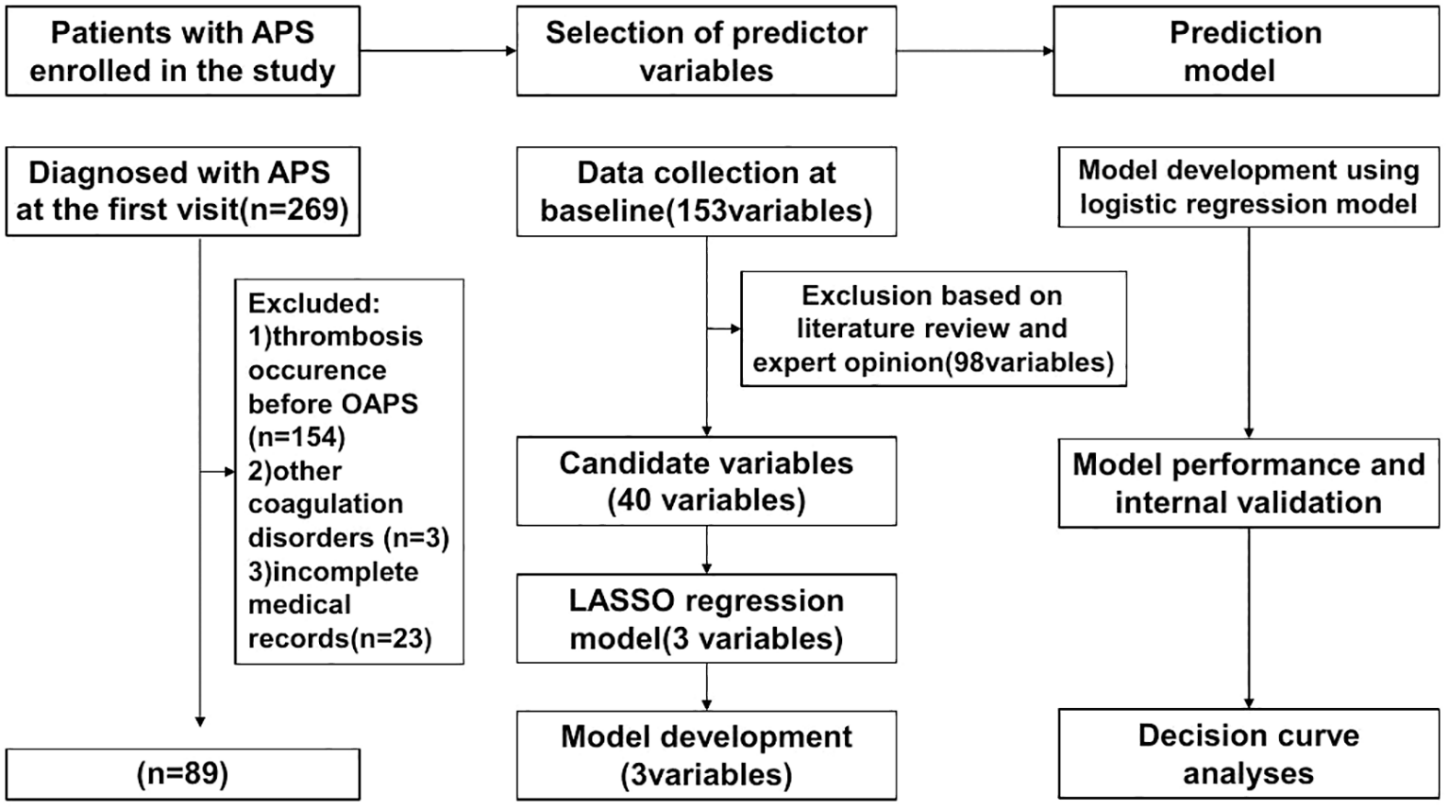
Flowchart for constructing the first occurrence of thrombosis in the OAPS prediction tool.

This study retrospectively included 269 consecutive APS patients admitted to the First Affiliated Hospital of the Fourth Military Medical University (Xijing Hospital) from July 2008 to July 2022. Among them, 115 were OAPS patients with a history of obstetric events only (no previous thrombotic events). All participants met the 2006 Sydney APS classification criteria or the non-criteria OAPS classification (1, 5). Exclusion criteria:1) thrombotic events prior to the onset of OAPS; 2) presence of other coagulation disorders, such as severe liver disease and malignancy; 3) patients with follow-up <1 year; 4) incomplete medical records. Follow-up time to the endpoint was calculated from the baseline to the date of diagnosis of thrombosis or to the last follow-up visit before the end of the study period (31 July 2023) for patients who did not develop thrombosis. Follow-up was censored at the time of the patient’s last visit if the patient died of other causes or was lost to follow-up. The final population eligible for thrombosis risk analysis included 89 patients (**Figure 1**). The study was conducted in accordance with the Declaration of Helsinki and good clinical practice.

Before developing the predictive model, we analyzed previous research and clinical data to identify potential factors influencing the outcome. We also incorporated our insights to identify unstudied factors. The variables include: 1) demographic characteristics such as age, gender, ethnicity, and education level; 2) past medical history, including autoimmune diseases (e.g., systemic lupus erythematosus, Sjögren’s syndrome), hypertension, diabetes, hyperlipidemia, and risk factors like smoking and alcohol consumption; 3) hospital laboratory indicators, such as blood tests, biochemical tests, coagulation function, and autoantibody series; 4) imaging indicators, including CT, MRI, and ultrasound; 5) clinical manifestations, including specific circumstances related to thrombosis and adverse pregnancy during disease onset; and 6) medication usage, such as long-term low-dose aspirin, low-molecular-weight heparin, and hydroxychloroquine sulfate. Demographic and clinical information at baseline were collected by trained researchers from electronic medical records. Patients were followed up through telephone interviews or outpatient visits to monitor their actual medication use and vascular thrombotic events. They were required to be followed up for at least one year. Patients were fully evaluated and documented every 3 to 12 months.

Hypertension was defined as having high blood pressure or using antihypertensive medication at two or more random time points. Diabetes mellitus was defined as having two or more fasting blood glucose levels > 7.0 mmol/L or using insulin or oral hypoglycemic agents. Smoking status was determined by self-reporting of tobacco consumption. Serum total cholesterol and HDL cholesterol levels were determined using standardized enzymatic methods and interpreted based on current threshold values.

### 2.2 Detection of aPLs

Plasma and serum samples were collected from patients and all aPLs tests were performed in our laboratory. The aPLs profile includes lupus anticoagulant (LA), anti-β2 glycoprotein I antibody (anti-β2GP1), and anticardiolipin antibody (aCL). Autoantibodies aCL and anti-β2GP1 were detected by ELISA. In our study, we did not conduct separate tests for the IgM and IgG subclasses of aCL and anti-β2GPI antibodies due to limitations in the hospital’s testing methods. Therefore, according to our laboratory’s reference interval, a positive result is defined as: aCL > 40 IU/mL and aβ2GPI > 40 RU/mL. Plasma samples were tested for the presence of LA according to the criteria recommended by the ISTH Lupus Anticoagulant/Antiphospholipid Dependent Antibody Subcommittee. Plasma sample was considered positive for LA if the Dilute Russell’s Viper Venom Test (dRVVT) ratio was > 1.2 (6). Only tests for aPLs diagnosed at least 12 weeks apart were considered positive.

### 2.3 Assessment of thrombosis

Thrombosis was diagnosed by two experienced immunologists based on clinical presentation and imaging findings (magnetic resonance imaging or computed tomography angiography or vascular ultrasound). Arterial thrombotic events included stroke, myocardial infarction, and arterial occlusion, were confirmed by computed tomography (CT), magnetic resonance imaging, conventional angiography, or vascular ultrasound. Venous thrombosis was defined as deep vein thrombosis and pulmonary thrombosis, and confirmed through CT scan, angiography or vascular ultrasound.

### 2.4 aGAPSS score

The aGAPSS was calculated by summarizing the corresponding risk factors: score 1 for arterial hypertension, score 3 for hyperlipidemia, score 4 for ab2GPI, score 4 for LAC, and score 5 for aCL.

### 2.5 Statistical method

The methods in this study were followed the Transparent Reporting of a Multivariable Prediction Model for Individual Prognosis or Diagnosis (TRIPOD) statement. Continuous variables are reported as medians (interquartile range [IQR]) and categorical variables as frequencies. Statistical analyses were performed using the Mann-Whitney U test, Fisher exact test or chi-squared test, as appropriate. P values less than 0.05 were considered significant. Least absolute shrinkage and selection operators (LASSO) were used to select the most predictive among the candidate variables. Based on these independent predictors, a nomogram was constructed and the area under the receiver operating characteristic (AUROC) curve was plotted to evaluate and compare the discriminative power of the nomogram with that of the aGAPSS. The AUC was calculated to compare the accuracy of the model parameters between different cut-off values.

Internal cross-validation of the predictive model was performed using the bootstrap method. The predictive performance of the model was assessed using Harrell’s consistency index (C-index) and calibration curves to validate model discrimination and calibration. Decision curve analysis was then used to assess the clinical benefit of our model. The two-sided P values for all statistical tests were < 0.05 and the differences were statistically significant. All statistical analyses were performed using IBM SPSS Statistics (version 25.0) and R software (version 4.3.1).

The main steps on developing a clinical prediction model are illustrated in the flowchart (**Figure 1**): First, initial variables were determined by combining literature research and clinical experience. Then, the predictive factors were identified through LASSO regression. Using these predictive factors, we created the prediction model and visualized it with a nomogram. Subsequently, the model was validated for performance and compared with the efficacy of previous relevant models.

## 3 Result

### 3.1 Baseline characters of the population

During the study period, there were a total of 269 patients initially identified as APS. According to the exclusion criteria, 89 patients were ultimately retained for analysis, with 154 patients were excluded if they had only thrombosis or thrombosis prior to the onset of OAPS, 3 patients were excluded due to other coagulopathies (2 with malignancy and 1 with severe liver disease), and 23 patients were excluded due to incomplete medical records. The average onset age of 27.34 ± 3.62 years.

During a mean follow-up of 3 years, 29 cases (32.58%) of patients with OAPS developed a new thrombus. We compared the baseline demographics, clinical characteristics and laboratory tests between those thrombotic patients with non-thrombotic patients (**Table 1**).

**Table 1:** Demographic and clinical characteristics of OAPS patients in the thrombotic vs non-thrombotic group.

Variables	Total (n = 89)	thrombotic (n = 60)	Non- thrombotic (n = 29)	p
Onset age,years	27.34 ± 3.62	27.73 ± 3.49	26.52 ± 3.81	0.154
Follow-up duration, years	3 (1, 6)	3 (1.19, 4.25)	3 (1, 9)	0.578
SLE, n (%)	35 (39)	19 (32)	16 (55)	0.058
nonstandard, n (%)	38 (43)	20 (33)	18 (62)	0.019
HP, n (%)	19 (21)	9 (15)	10 (34)	0.068
HLP, n (%)	20 (22)	9 (15)	11 (38)	0.031
ab2GP1, n (%)	62 (70)	38 (63)	24 (83)	0.105
LAC, n (%)	32 (36)	18 (30)	14 (48)	0.148
aCL, n (%)	52 (58)	30 (50)	22 (76)	0.037
morbidfrequency, n (%)				0.572
1	22 (25)	16 (27)	6 (21)	
2	40 (45)	26 (43)	14 (48)	
3	14 (16)	11 (18)	3 (10)	
4	10 (11)	6 (10)	4 (14)	
5	2 (2)	1 (2)	1 (3)	
6	1 (1)	0 (0)	1 (3)	
heart, n (%)	27 (30)	14 (23)	13 (45)	0.069
plt, Median (Q1,Q3)	134 (84, 203)	159.5 (121.5, 222)	86 (61, 114)	< 0.001
PLT125, n (%)	42 (47)	16 (27)	26 (90)	< 0.001
IgG, n (%)	21 (24)	15 (25)	6 (21)	0.855
IgM, n (%)	6 (7)	4 (7)	2 (7)	1
IgA, n (%)	11 (12)	7 (12)	4 (14)	0.744
C3, n (%)	45 (51)	26 (43)	19 (66)	0.083
C4, n (%)	44 (49)	24 (40)	20 (69)	0.02
aPLs2o3, n (%)	43 (48)	21 (35)	22 (76)	< 0.001
PLDAorLMWH, n (%)	70 (79)	52 (87)	18 (62)	0.017
DLDAorLMWH, n (%)	43 (48)	32 (58)	8 (28)	0.013
IVIG, n (%)	22 (25)	16 (27)	6 (21)	0.726
Phormonotherapy, n (%)	51 (57)	34 (57)	17 (59)	1
Pimmunosuppressor, n (%)	34 (38)	21 (35)	13 (45)	0.508
PHCQ, n (%)	59 (66)	41 (68)	18 (62)	0.729
mono1, n (%)	25 (28)	20 (33)	5 (17)	0.183
neutro1, n (%)	32 (36)	25 (42)	7 (24)	0.168
lym1, n (%)	33 (37)	16 (27)	17 (59)	0.007
mpv1, n (%)	29 (33)	17 (28)	12 (41)	0.322
pdw1, n (%)	44 (49)	30 (50)	14 (48)	1
pt1, n (%)	26 (29)	14 (23)	12 (41)	0.132
aptt1, n (%)	42 (47)	26 (43)	16 (55)	0.411
fib1, n (%)	49 (55)	37 (62)	12 (41)	0.115
pta1, n (%)	60 (67)	44 (73)	16 (55)	0.141
inr1, n (%)	39 (44)	30 (50)	9 (31)	0.144
ddi1, n (%)	53 (60)	38 (63)	15 (52)	0.415
fdp1, n (%)	14 (16)	8 (13)	6 (21)	0.371
aGAPSS, Median (Q1,Q3)	8 (4, 10)	5 (4, 9)	10 (8, 13)	< 0.001
aGAPSSover10, n (%)	28 (31)	13 (22)	15 (52)	0.009

### 3.2 Development of predictive models

#### 3.2.1 Selection of predictor variables

**Figure 2:**
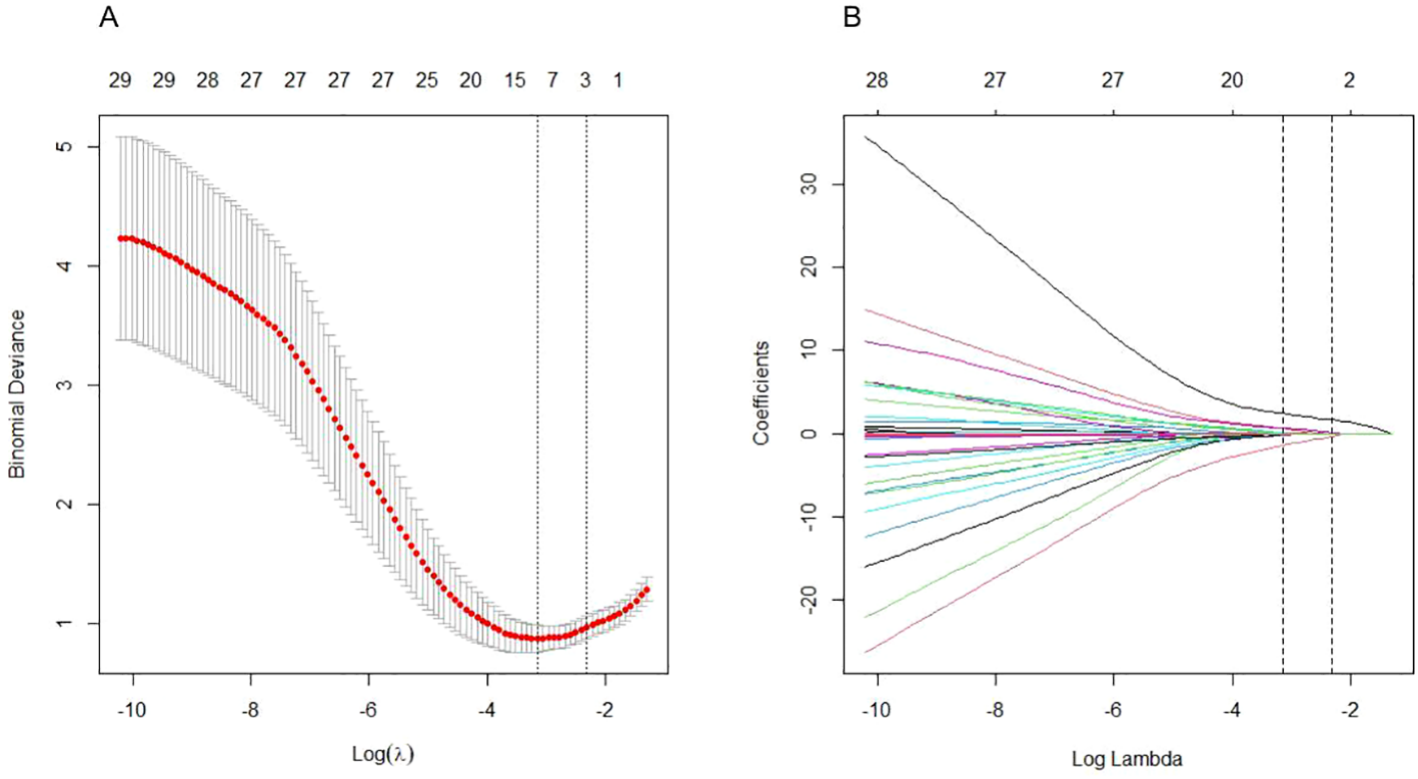
Clinical feature and laboratory parameter selection using the LASSO logistic regression model. **(A)** Optimal parameter (lambda) selection by LASSO used tenfold cross validation via minimum criteria. The average number of predicted variables is expressed as a number along the upper x-axis. The average deviation of each model is represented by a red dot, and the upper and lower limits of the deviation are represented by vertical lines passing through the red dot. The best value of lambda is defined by a vertical black line (λ=0.054). **(B)** LASSO coefficient profiles of the variables plotted against the log(lambda) sequence. Drawing vertical lines by optimum lambda values of three nonzero coefficients through tenfold cross-validation.

After data collection, we found 98 potentially correlated variables. To enhance clinical practice, it is vital that the chosen parameters are readily accessible. Consequently, after discussions among the three corresponding authors, we eliminated variables like lymphocyte subsets and cytokines, focusing on 40 potential candidate variables (**Figure 1**). To avoid overfitting, we utilized the Least Absolute Shrinkage and Selection Operator (LASSO) logistic regression to choose the most predictive variables from the 40 potential candidate variables preselected based on expert opinion (**Figure 2**). Before including them in the final model, the regression model was built by considering the number and appropriateness of the predictor variables selected. Based on the number of variables displayed by lambda.1se, the three predictor variables were selected to develop the model and estimate the coefficients associated with each significant predictor variable.

#### 3.2.2 Construction of nomogram

**Figure 3:**
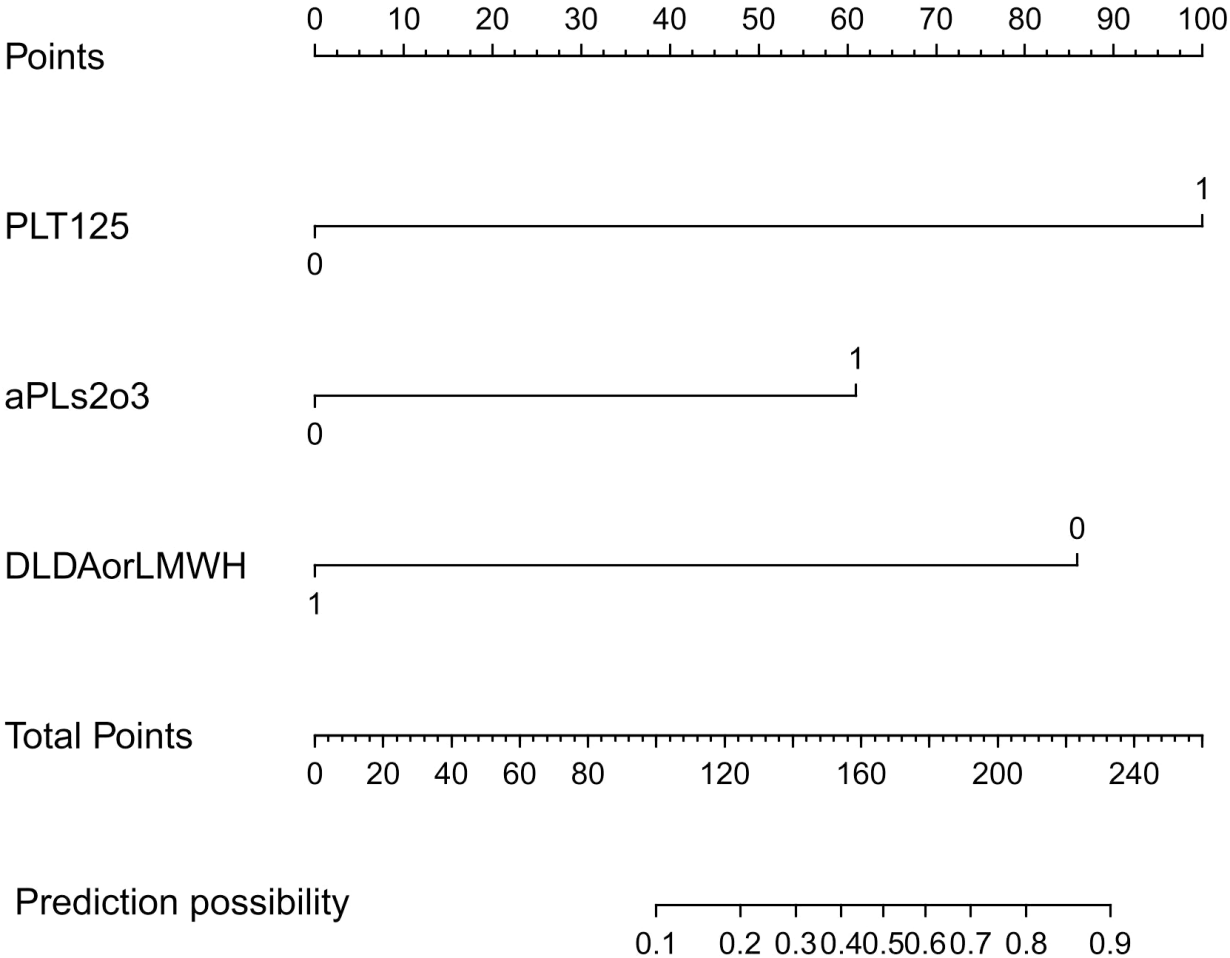
Plotting of nomogram according to predictors. This model was developed with PLT125, aPLs2o3, DLDAorLMWH. The scale of the line segment corresponding to each variable in the prediction model indicates the possible value range of the variable, and the length of the line segment indicates the influence of the factor on the outcome event. Point represents the individual score corresponding to each variable under different values, and the total score is obtained by adding the individual scores of all variables. Risk represents the risk of the first occurrence of thrombosis in patients with OAPS. PLT125: blood platelet less than 125×10^9^/L; aPLs2o3: two or three aPLs are positive; DLDAorLMWH: patient receive LDA or LMWH after delivery.

Based on these independent predictors, we constructed a predictive nomogram for first-time thrombosis in OAPS using the rms package of the R software (**Figure 3**). A nomogram is a graphical tool used to visually represent a predictive model, illustrating the relationships between variables. It is derived from the results of multifactorial regression analysis. Each variable is plotted alongside its corresponding score on a unified scale. In clinical practice, doctors can use the nomogram by determining the values of the predictors for each patient and following these steps: First, assign scores to each predictor’s value based on the nomogram’s scale. Next, sum all the scores to obtain a total score. Finally, use this total score to find the corresponding risk prediction value on the nomogram. For each patient diagnosed with purely OAPS, we summed the scores from the three identified risk factors. The probability of the patient’s risk of a thrombotic event was then determined based on the “total points” axis of the nomogram. We have provided an example of the clinical application of the nomogram in the **Supplementary Material** (**Supplementary Figure S1**), demonstrating how to conduct risk assessment based on the specific conditions of a patient.

#### 3.2.3 Construction of risk scoring and network calculator

A risk score for the first thrombosis in patients with OAPS was derived from the coefficients of the logistic model. We calculated the probabilities and 95% confidence intervals of the logistic model using the following formula. The risk of the first thrombosis in an individual patient with OAPS can be calculated using the following formula: prognostic index = 3.3058×PLT125 + 2.0162×aPLs2o3 - 2.8393×DLDAorLMWH. All variables were coded as dichotomous. Where DLDAorLMWH was negatively correlated with thrombosis, the other two predictors were positively correlated.

### 3.3 Model performance evaluation and internal validation

**Figure 4:**
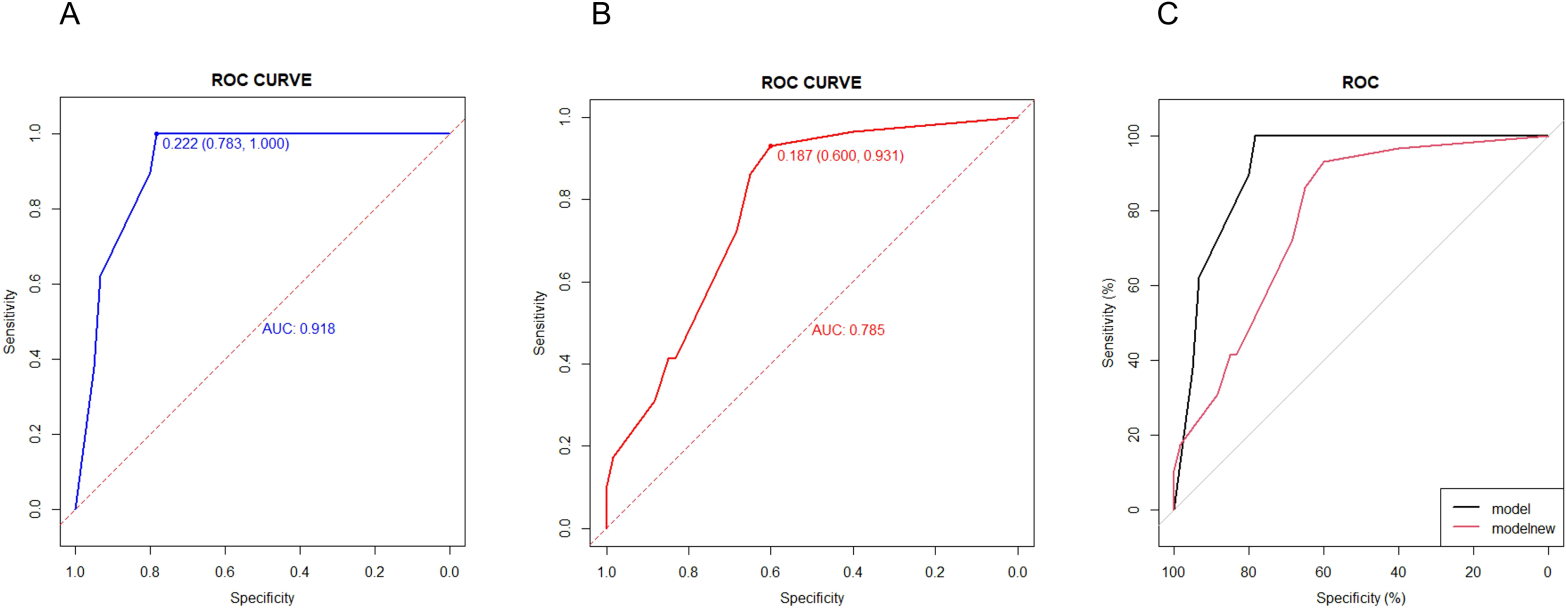
Efficiency and validation of the disease diagnosis model. **(A)** ROC of new model in OAPS patients (ROC of the first occurrence of thrombosis in patients with OAPS). **(B)** ROC of aGAPSS model in OAPS patients. **(C)** ROC of new model and aGAPSS model in OAPS patients.

**Figure 5:**
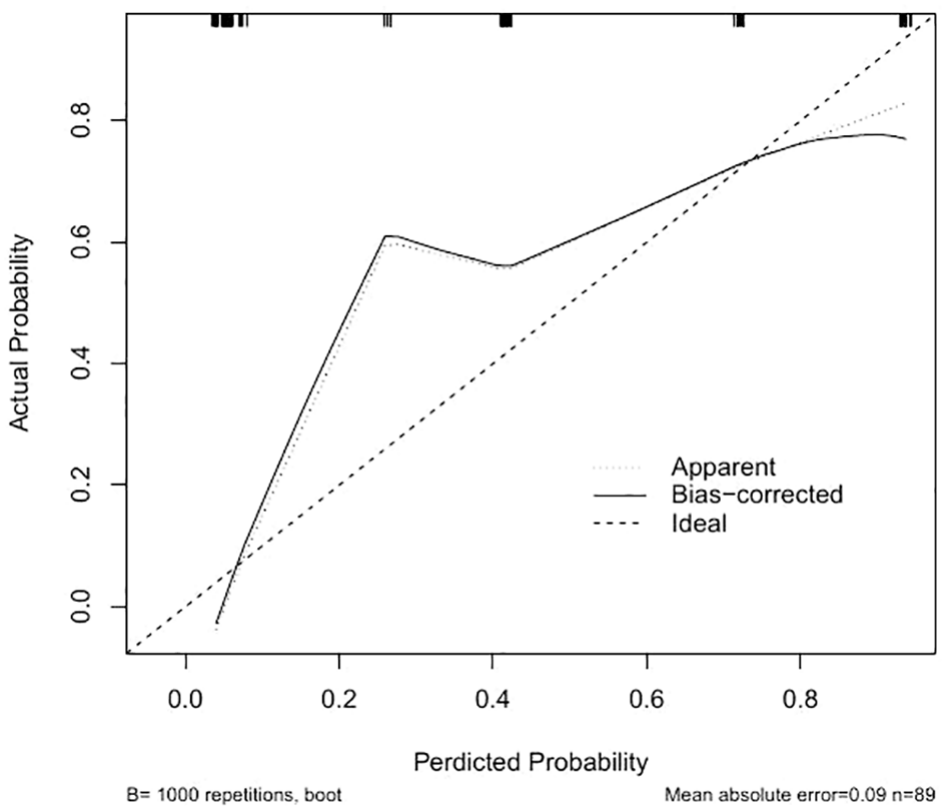
Creation of calibration plots of the new model. “Apparent” is the uncalibrated prediction curve, “Bias-correctrd” is the calibrated prediction curve, and “Ideal” is the standard curve, which represents the perfect prediction of the ideal model. The Y-axis represents the actual prevalence of the first occurrence of thrombosis in patients with OAPS. The X-axis represents the predicted risk of the first occurrence of thrombosis in patients with OAPS in the cohort.

To assess and contrast the identification of the new model with aGAPSS, the area under the receiver operating characteristic (AUROC) curve was plotted. The AUC of the new model was greater than that of aGAPSS [0.9181(95% CI, 0.8634-0.9728) vs. 0.7848 (95% CI, 0.6899-0.8796), P<0.001], indicating that the new model has better discriminatory power (**Figure 4**). To ensure accurate prediction of column-line plots, calibration curves were created by plotting the observed probabilities against the predicted probabilities of the Noetherian plots. The two-sided p-values for all statistical tests were less than 0.05 and the differences were statistically significant (**Figure 5**). The Brier score of the model was 0.107. The comparison between predicted risk and observed outcomes is shown in 200 Bootstrap of calibration plots. Hosmer-lemeshow test is 0.083.

### 3.4 Net benefit of predictive modelling

**Figure 6:**
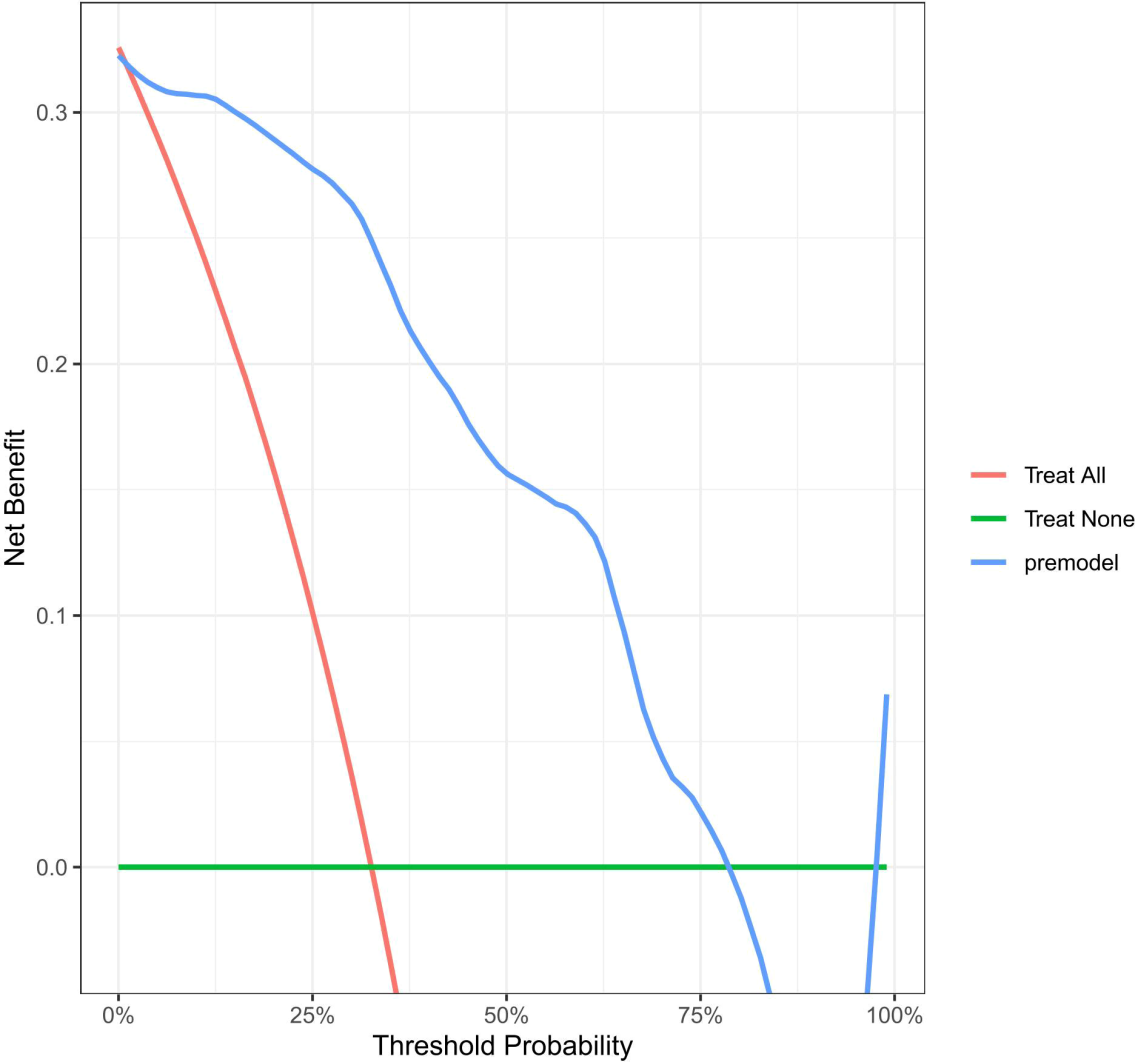
Decision curve for the model in predicting the first occurrence of thrombosis in patients with OAPS. Standard net benefit (y-axis) and risk threshold (x-axis) formed the coordinate system. The blue line represented our model, red line represented the assumption that all the people had occurred thrombosis after delivery, and green line represented the assumption that all the people hadn’t occurred thrombosis after delivery.

The potential net benefit at different probability thresholds was assessed and compared through decision curve analysis (DCA) using the rmda software package (**Figure 6**). To compared screening based on whether all patients with OAPS would experience a thrombotic event or not, the DCA was performed. The curves from the predictive model showed positive net benefits for probability thresholds between 1% and 78%. This suggests that using the model to inform clinical decisions will lead to better outcomes for any decision in this range.

## 4 Discussion

The retrospective cohort study conducted at our center provides data to explore risk factors for the first-onset thrombosis in patients with OAPS, and to develop appropriate clinical prediction models. Our purpose is to develop a risk prediction model that is user-friendly for both clinicians and patients. In the internal validation of the study cohort, the model demonstrated satisfactory performance with an AUC-based accuracy of 0.9181. After appropriate calibration, the resulting nomogram was effective in predicting the risk of future thrombotic events in patients with OAPS alone. Decision curve analysis demonstrated that if the threshold probability of patients is between 1% to 78%, using this model to predict thrombosis events would provide more benefits compared to either the treat-all strategy or the treat-none strategy. The three variables required to calculate the risk of time to thrombosis in our prediction model are typically available at the time of the visit, and the user-friendly design of the network calculator facilitates its semless integration into routine practice. To our knowledge, this is the first clinical prediction model currently available for initial thrombotic events in APS patients with obstetric-only events. If a patient’s estimated risk of a thrombotic event is low, clinicians may choose to follow up; however, if the risk is high, clinicians can be guided to screen for thrombosis in conjunction with the patient’s clinical presentation.

Our study revealed that 32.58% (29/89) of patients with OAPS experienced thrombotic events during a median follow-up of 3 years. De Jesús, G. R. et al. conducted a multicenter retrospective study using the APS Clinical Trials and Consortium for International Networking (APS ACTION) clinical database and knowledge base. The study revealed that 63% (47/74) of women with OAPS experienced a thrombotic event after the initial obstetric onset, with an average time of 7.6 ± 8.2 years. A younger age at the diagnosis of OAPS, additional cardiovascular risk factors, superficial venous thrombosis, valvular heart disease, and multiple aPL positivity all increase the risk of a first thrombosis after pregnancy morbidity (7). The risk factors in this article did not include indicators such as complement, platelet, lymphocyte counts, and preconception pregnancy and perinatal medications, which recent studies have shown may be associated with the development of OAPS. In addition, the rate of thrombosis in this study was higher than in our study, which may be related to the longer follow-up period. Silver et al. first reported that 15.7% of obstetric APS patients had thrombosis during a median follow-up of 3 years (8). The percentage was lower than in our study, possibly due to the inclusion of APS patients with pre-existing thrombosis before the study, and a higher percentage of prolonged anticoagulant use in the postpartum period compared to our study. A retrospective case-control study was conducted in 2005, 141 women with OAPS were matched with 141 women with idiopathic recurrent miscarriage. Most of the patients in the study reported that thrombosis occurred in the brain and were predominantly arterial (9). Our study also suggests that patients with OAPS have a higher risk of developing arterial thrombosis, particularly arterial cerebrovascular events. Therefore, it is recommended that patients be promptly evaluated for cerebrovascular events when they present with symptoms such as dizziness, headache, and numbness of the limbs.

The GAPSS is an internationally recognized thrombosis risk prediction score that comprises six items. The score was initially developed and validated in a group of patients with systemic lupus erythematosus and subsequently in patients with APS without SLE (2). In the aGAPSS, anti-APS/PT was removed to enhance clinical applicability. The clinical utility of the aGAPSS in assessing thrombosis risk in various clinical settings has been described and validated. Baseline characteristics of different patient groups explain the variations in aGAPSS thresholds. Although there is no precise cut-off value that distinguishes between low and high risk, aGAPSS may be a useful tool for assessing new thrombotic events in patients with APS and for guiding pharmacologic therapy in high-risk patients (3, 10–13). In our retrospective study, we found that patients with thrombosis had higher aGAPSS values compared to patients without thrombosis.

However, aGAPSS has limited ability to assess obstetric events (14), possibly because the pathogenesis of OAPS differs from the pathophysiological mechanisms of TAPS (15–17). Due to the specificity of OAPS, which involves obstetric pathophysiologic mechanisms in addition to the common risk factors for thrombosis, our study also included variables related to adverse pregnancies. In our study, the baseline variables included numerous risk factors that previous studies have suggested may be associated with thrombotic recurrence in patients with APS (18–21). By using LASSO regression, it was confirmed that thrombocytopenia, multiple aPLs, and the use of anticoagulants and antiplatelets after delivery was confirmed to be the strongest risk factors predicting thrombotic events in patients with OAPS. The final selection of predictors was confirmed by expert opinion in terms of clinical validity, feasibility, and applicability, and internally validated for stability. After comparison, the AUC of our new model predicting the occurrence of thrombosis in OAPS (0.918) was higher than the AUC of aGAPSS (0.785), and the difference was statistically significant.

In 2023, the ACR/EULAR published new classification criteria in the form of a scoring system that categorizes additional weighted criteria into six clinical and two laboratory domains (4). Several weighted criteria from the new 2023 version of the classification criteria were incorporated into the variables included in our study. Among the final predictors of thrombosis risk obtained from the analysis of the study results, thrombocytopenia is also a new element that appears in the new classification criteria. Platelets are small, anuclear, multifunctional blood cells that play an important role in the pathogenesis of antiphospholipid syndrome (22, 23). Platelets play a crucial role in the development of APS by regulating coagulation, thrombosis, inflammation, and innate immunity. They also interact with components of the immune systems such as neutrophils and lymphocytes (24, 25). A study by X, Zeng et al. confirmed the correlation between PDW and thrombotic events in patients with APS, supporting the theory that platelet activation is an important mechanism of thrombosis in patients with APS (26). B, Artim-Esen’s study showed that among aPLs-positive patients, those with a low platelet count were more likely to develop thrombosis (27).

This retrospective study focuses on patients diagnosed with OAPS from July 2008 to July 2022 in our hospital. At that time, the new classification criteria for APS had not yet been published, and the 2006 Sydney criteria were primarily used in clinical practice. We reviewed the new criteria and reassessed the patients in our study. Among the 89 patients previously diagnosed with purely OAPS, 61 patients (68.54%) met the new OAPS classification criteria. To assess compliance rates for the old and new classification criteria, we focused on adverse pregnancy events in patients who did not meet the new criteria. These events included: early fetal deaths from 10 weeks to 15 weeks and 6 days(2/89), as well as fetal deaths from 16 weeks to 33 weeks and 6 days without severe preeclampsia or placental insufficiency (5/89). According to the new criteria, each of these items is assigned a value of 1 point, while a minimum score of 3 points is required for the clinical domain. Based on our inclusion criteria, 21 patients with non-standard obstetric antiphospholipid syndrome were also analyzed (21/89). Hence, they do not comply. It is crucial to conduct further research to compare the performance of the old and new classification criteria in clinical settings and to validate their effectiveness across various populations.

Increasing evidence suggests that each aPL is associated with a high risk of thrombosis, and complete antiphospholipid antibody profiling may better identify patients at risk than a single test. Therefore, risk stratification based on the number of positive tests has been proposed (18, 20, 21), and our data support this approach. Pengo et al. analyzed data from 618 consecutive patients over 6 years and found that triple-positive patients had the highest risk of thrombosis. They were also associated with a high risk of pregnancy morbidity, which was not seen in single-positive aPL (28). A prospective cohort study in Finland showed that double- or triple-positive aPLs was a risk factor for future thrombotic events, especially in individuals with underlying autoimmune disease, while being single-positive did not appear to increase the risk of thrombosis (29). Therefore, the combined use of aPL testing should be considered when discussing the risk of thrombosis in patients with OAPS.

According to the guidelines, women who receive LDA and prophylactic doses of LMWA prenatally should be treated with low-molecular-weight heparin continuously for 6 weeks postpartum (30, 31). Combined antiplatelet and anticoagulant therapy reduces the rate and prolongs the time to thrombotic recurrence in patients with APS associated with arterial thrombosis compared with monotherapy (30–34). The puerperal period carries an increased risk of thrombosis. Our research recorded thrombotic events in four patients during the puerperium, including two cases of cerebral infarction and two cases of deep vein thrombosis in the lower limbs. Our data indicate that patients with Obstetric Antiphospholipid Syndrome (OAPS) are at a higher risk of thrombosis during the puerperal period compared to the general population (35, 36). In our study, only 48% of patients were treated with LDA and/or LMWA in the postoperative period, which may be one of the reasons for the higher rate of thrombosis than in other studies (37). This further emphasizes the importance of thrombosis prevention in OAPS patients during the puerperium. We believe that these data analyses can provide valuable reference for clinical practice. It also suggests that rheumatologists need to communicate more with obstetricians and gynecologists about the latest academic advances in postpartum treatment and follow-up of OAPS (38).

The predictive model we developed in this study has some limitations: 1) this is a single-center, retrospective study with a small sample size, which may limit the generalizability of the prediction model to other regions. Therefore, further large sample studies are needed to confirm our model and measure its performance, especially its applicability to other regions and ethnicities; 2) the results of this study were evaluated through internal validation of the model and not have not been validated by external data sets. Multicenter external validation in other regions should be completed at a later date to further assess the performance of the model; 3) Other predictors, such as cytokine levels and lymphocyte subpopulation counts, were not included in the current study due to the high level of missing data. These variables should be investigated by including them in future prospective studies; 4) The 2023 ACR/EULAR APS classification criteria emphasize the importance of distinguishing between the IgG and IgM isotypes of aCL and anti-β2GPI antibodies (4). Our study could not perform separate tests for the IgM and IgG isotypes of aCL and anti-β2GPI antibodies because of hospital testing limitations. This limitation may affect our sensitivity in predicting thrombotic events and decrease the positive predictive value. We will implement modified testing methods to differentiate antibody isotypes in the future. We will establish medium and high titer thresholds for aCL and anti-β2GPI antibodies in the local population. These thresholds will be incorporated into our upcoming observational clinical studies. Additionally, we will conduct additional analyses to evaluate how antibody isotypes and the number of positive results influence the risk of thrombosis and pregnancy complications in APS patients.

Our study also has several advantages. Firstly, it was more targeted than previous studies, focusing solely on a subset of OAPS patients with only obstetric events. Secondly, we aimed to create a screening tool for clinical use. This predictive model mainly incorporates available clinical information and routine laboratory test results as variables. Thirdly, we excluded variables such as erythrocyte sedimentation rate or C-reactive protein level, which may change within a short period of time. We also converted most of the numerical variables to dichotomous variables, thereby reducing inconsistent observations of numerical fluctuations during the follow-up period.

In conclusion, we developed a risk prediction model using routine clinical assessments. Three risk variables included PLT125, aPLs2o3 and DLDAorLMWH constructed a prognostic scoring index, and this new tool may effectively predict the future risk of thrombotic events in OAPS patients with a history of obstetric events only (without prior thrombotic events). Based on the net benefit and predictive probability thresholds, we recommend annual screening for high-risk patients.

## Data Availability

The original contributions presented in the study are included in the article/**Supplementary Material**. Further inquiries can be directed to the corresponding authors.
