# Supplementary material for: Development of a risk prediction model for the first occurrence of thrombosis in patients with OAPS
Source: Front Immunol. 2024 Oct 4;15:1459548. doi: 10.3389/fimmu.2024.1459548 (PMC11486719; doi:10.3389/fimmu.2024.1459548)
Supplement: Supplementary file 1 [file DataSheet1.docx]

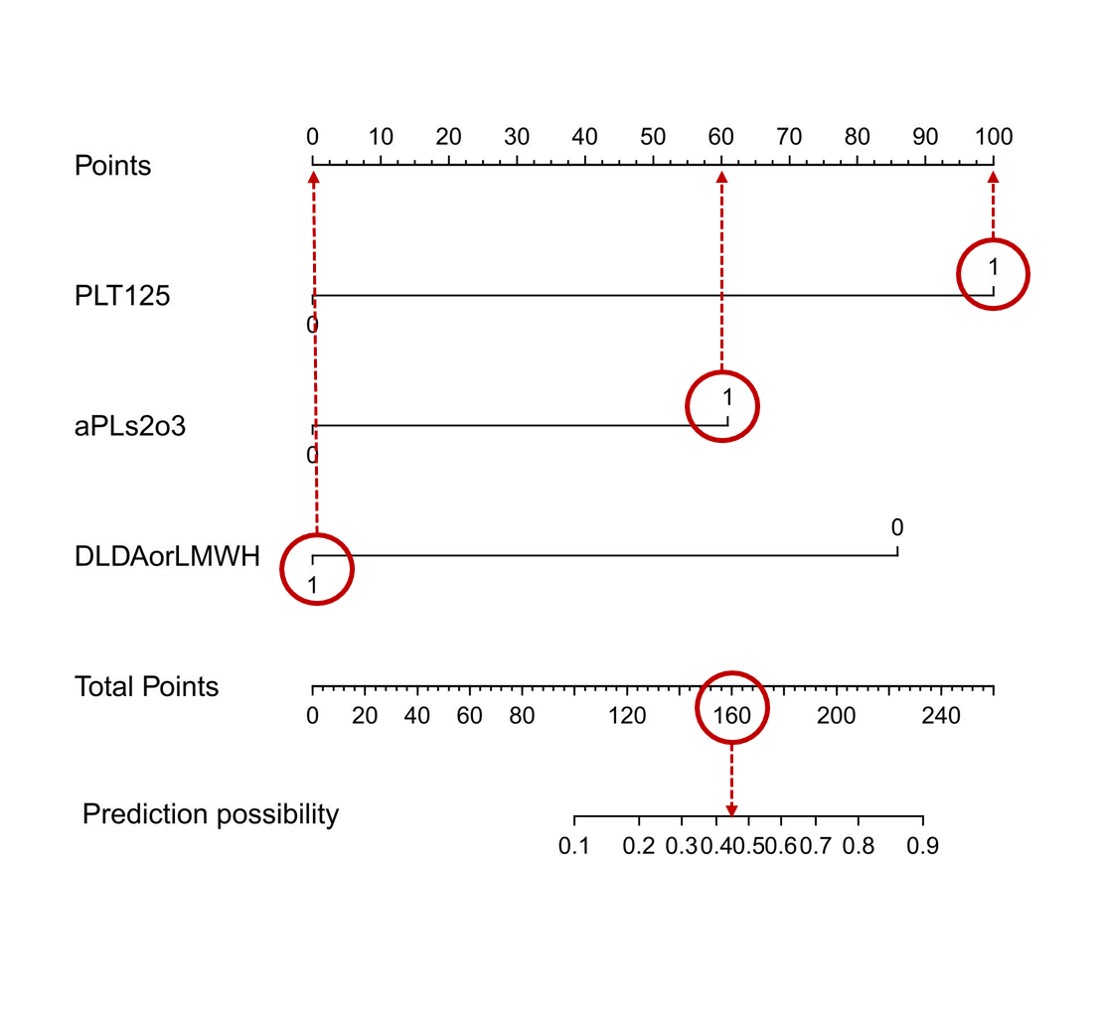


Supplementary Figure1: For instance, if a purely OAPS patient comes to the outpatient clinic for follow-up, and her platelet retest is 110×10^9^/L, then the "PLT125" column will be awarded 1 point, and the corresponding points will be 100 points; During previous tests for antiphospholipid antibodies, both anti-β2GPI and anticardiolipin antibodies were both positive with high titers, 1 point was awarded in the column of "aPLs2o3", and the corresponding points were 60 points; The patient was regularly treated with low molecular weight heparin during the puerperium, and the score in the column "DLDAorLMWH" was 0, and the corresponding points were 0. The total score of the three items is 160 points, and a score of 160 on the "Total Points" corresponds to a "Prediction possibility" of 45%, indicating that the patient has a 45% chance of developing blood clots in the future. These results indicate that the doctor should monitor the patient for symptoms of thrombosis and schedule regular blood clotting tests during future examinations.
